# Commentary: Reaching beyond words: supporting self-care through visual health education

**DOI:** 10.1177/17449871251370626

**Published:** 2025-10-23

**Authors:** Pernilla Garmy

**Affiliations:** Professor, Kristianstad University, Sweden

**Commentary to:** Living well guide: a nurse-led development and validation of a printed resource for cardiometabolic care (Macêdo et al. 2025)

There is a well-known gap between what we know we should do for our health – and what we actually do. When it comes to healthy lifestyle behaviours, this gap is particularly visible. Despite good intentions, access to care and well-established knowledge, patients often struggle to change long-standing habits. This is why creative, accessible, and person-centred educational tools are urgently needed.

In their methodological study, Macêdo et al. (2025) presented the development and validation of an educational resource aimed at people living with cardiometabolic diseases. The printed resource combines comic-style illustrations with brief, easy-to-understand messages on topics such as nutrition, hydration, physical activity, sleep, stress, medication use, smoking cessation and alcohol reduction. Importantly, the resource also explains what cardiometabolic diseases are – something many patients find difficult to articulate or fully understand.

From my own experience as a nurse and educator, I know that there is no single educational format that works for all patients. Some people benefit from verbal dialogue, others prefer detailed written materials. But for many, visual aids are a crucial complement. Pictures can clarify, engage and motivate – especially when literacy is limited or when energy and focus are low due to illness, stress, or comorbidities.

The fact that this resource is printed – not digital – also matters. A physical object can be held, browsed at one’s own pace, and brought along to consultations. It does not require a smartphone, internet access, or digital skills; which may be barriers for some groups. At the same time, digital tools may be more effective in other contexts. The point is: healthcare professionals need a flexible set of educational tools, adapted to the needs, abilities, and preferences of each individual.

The use of visual materials in health communication is supported by robust evidence. A systematic review and meta-analysis by Schubbe et al. (2020), including 54 randomised controlled trials, showed that pictorial health information improves understanding, memory, and adherence to health advice – especially among people with limited health literacy. The authors concluded that icons and images with few words may be particularly effective, which aligns well with the design of the serial resource developed by Macêdo et al. (2025).

The resource was developed in four distinct phases. Firstly, a systematic literature review was conducted to identify key themes in cardiometabolic health promotion. This was followed by a qualitative study, including interviews with nine patients living with cardiovascular disease. Their most important information needs revolved around diet, sleep hygiene, physical activity, necessary lifestyle changes, and medication use. These topics formed the foundation of the resource’s content.

The next phase involved content development and visual design, including collaboration with a professional graphic designer. Importantly, the material underwent two rounds of validation: first by 22 healthcare professionals – mainly nurses – with expertise in cardiometabolic care and patient education, and then by 22 patients representing the target audience. Both groups rated the resource as relevant, understandable, and suitable for use in healthcare.

The final version of the educational serial album consists of 26 illustrated pages and features a recurring set of characters. Nurse Julia serves as the guide and educator, whereas Mr. John, Mrs. Rachel, Ana, and Alexander represent a diverse range of patient experiences. A friendly mascot – Heartie – adds an engaging and supportive tone. The characters appear in various everyday situations, helping readers relate to the content and see themselves in the narrative. This storytelling element, combined with clear messaging and familiar scenarios, turns the resource into more than just a source of information. It becomes a visual and emotional companion, capable of supporting reflection and behaviour change.

Educational technologies like this one play a key role in strengthening the nurse–patient relationship. They enable healthcare professionals to explain complex information in a relatable and engaging way, while also encouraging patients to take a more active role in their own care. For patients with cardiometabolic diseases – many of whom face multiple, long-term challenges – such tools can promote self-efficacy, adherence, and shared decision-making.

In summary, Macêdo et al. (2025) had created a resource that is co-designed with its target audience. It offers a concrete, validated tool to support health promotion and self-care – not only in Brazil but also potentially in many other settings where cardiometabolic disease burdens are high. As nurses, we must continue to explore and expand the use of visual, printed, and narrative-based materials in our practice. After all, if we want patients to make real, lasting lifestyle changes, we must first make our messages truly accessible, engaging, and meaningful.
